# Role of SYVN1 in the control of airway remodeling in asthma protection by promoting SIRT2 ubiquitination and degradation

**DOI:** 10.1186/s40659-023-00478-7

**Published:** 2023-12-02

**Authors:** Bing Dai, Si Liu, Wenxin Shen, Li Chen, Qianlan Zhou, Lina Han, Qinzhen Zhang, Lishen Shan

**Affiliations:** Department of Pediatrics, Shengjing Hospital of China Medical University, No. 36, Sanhao Street, Shenyang, 110004 China

**Keywords:** SYVN1, SIRT2, Ubiquitination, Endoplasmic reticulum stress, Epithelial-mesenchymal transition, Asthma

## Abstract

**Background:**

Asthma is a heterogenous disease that characterized by airway remodeling. SYVN1 (Synoviolin 1) acts as an E3 ligase to mediate the suppression of endoplasmic reticulum (ER) stress through ubiquitination and degradation. However, the role of SYVN1 in the pathogenesis of asthma is unclear.

**Results:**

In the present study, an ovalbumin (OVA)-induced murine model was used to evaluate the effect of SYVN1 on asthma. An increase in SYVN1 expression was observed in the lungs of mice after OVA induction. Overexpression of SYVN1 attenuated airway inflammation, goblet cell hyperplasia and collagen deposition induced by OVA. The increased ER stress-related proteins and altered epithelial-mesenchymal transition (EMT) markers were also inhibited by SYVN1 in vivo. Next, TGF-β1-induced bronchial epithelial cells (BEAS-2B) were used to induce EMT process in vitro. Results showed that TGF-β1 stimulation downregulated the expression of SYVN1, and SYVN1 overexpression prevented ER stress response and EMT process in TGF-β1-induced cells. In addition, we identified that SYVN1 bound to SIRT2 and promoted its ubiquitination and degradation. SIRT2 overexpression abrogated the protection of SYVN1 on ER stress and EMT in vitro.

**Conclusions:**

These data suggest that SYVN1 suppresses ER stress through the ubiquitination and degradation of SIRT2 to block EMT process, thereby protecting against airway remodeling in asthma.

## Background

Asthma is a common chronic inflammatory disease that involves in various cell types and multiple cytokines, along with airway hyperresponsiveness, inflammation and remodeling [1]. Epidemiological investigations suggest that asthma causes significant health issue and economic burden with the increasing incidence worldwide [2]. Chronic airway inflammation, injury and abnormal tissue repair contribute to an irreversible airway structural remodeling in asthma. It is characterized by mucus metaplasia, basement membrane thickening, angiogenesis, subepithelial fibrosis as well as smooth muscle cell hypertrophy and hyperplasia [3]. Airway remodeling in asthma is shown to be correlated with the prognosis of this disease [4]. Furthermore, epithelial-mesenchymal transition (EMT) is a critical pathophysiological process in which epithelial cells transform towards differentiated mesenchymal cells by losing epithelial cell polarity and the epithelial cell marker E-cadherin, as well as acquiring migration ability and the mesenchymal cell marker Vimentin. The occurrence of EMT is recognized to drive airway remodeling in asthma [5]. Therefore, studies on EMT are critical for exploring airway remodeling in asthma.

SYVN1 (Synoviolin 1), also called HRD1 or DER3, is an E3 ubiquitin ligase that promotes unfolded protein degradation via the ubiquitination-proteasome pathway, resulting in the inhibition of endoplasmic reticulum (ER) stress [6]. Recent studies have shown that ER stress is an important process that aggravates airway inflammation and remodeling [7, 8]. Inhibiting ER stress responses has been suggested to mitigate subepithelial fibrosis in chronic asthma [9], but its exact mechanism in asthma is yet to be clarified. In addition, we identify that the expression of SYVN1 is upregulated in asthmatic mice from the GEO dataset (Accession: GSE27066), suggesting possible implications of SYVN1 in the pathogenesis of asthma.

SIRT2 (Sirtuin 2) is a NAD-dependent histone deacetylase that is involved in the pathogenesis of asthma. For instance, accumulating studies have shown that SIRT2 exacerbates airway inflammation in allergic asthma [10, 11]. Pharmacological inhibition of SIRT2 with the specific inhibitor prevents OVA-induced lung fibrosis and inflammatory response in asthma [12]. Notably, Liu et al. have revealed that SIRT2 interacts with SYVN1 and is degraded by SYVN1 through the ubiquitination-proteasome system [13]. Thus, we speculated that SYVN1 might mitigate airway remodeling by promoting SIRT2 ubiquitination and degradation.

## Results

### SYVN1 is upregulated in lung tissues of OVA-induced mice

**Fig. 1:**
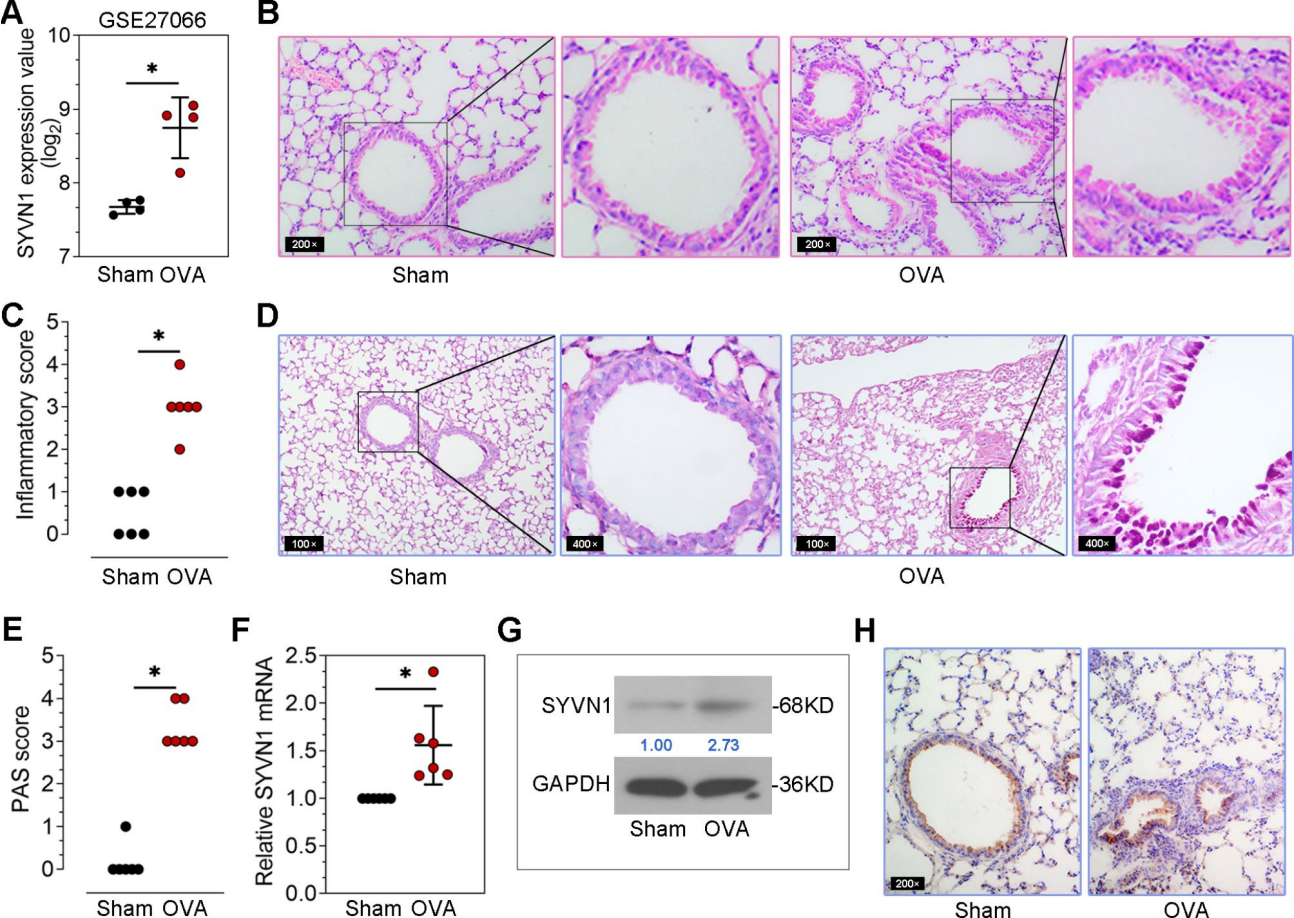
**SYVN1 is upregulated in lung tissues of OVA-induced mice**. (**A**), SYVN1 mRNA expression in lungs of asthmatic mice in GSE27066. (**B-C**), Representative images of the lung sections stained with H&E. The inflammatory infiltration was scored. (**D-E**), Representative images of the lung sections stained with PAS. PAS scores were quantified to evaluate the goblet cell hyperplasia. (**F-G**), Real-time PCR and Western blot analysis of SYVN1 mRNA and protein expression in the lungs. (**H**), Immunohistochemical examination of SYVN1 expression in the lung sections. *, *P* < 0.05

As shown in Fig. 1A, we identify that SYVN1 expression is upregulated in the lungs of OVA-induced mice from GSE27066 (https://www.ncbi.nlm.nih.gov/geo/query/acc.cgi?acc=GSE27066). In this study, OVA-induced asthmatic mice were performed. The pathological changes in lung tissues were evaluated using H&E and PAS staining. As shown in Fig. 1B-E, significant inflammatory cell infiltration and goblet cell hyperplasia were observed in the lung tissues of OVA-induced mice. Consistent with the results in GSE20766 (Fig. 1A), the increased SYVN1 expression was found at both the mRNA and protein levels in the lungs of OVA-induced mice (Fig. 1F-G). In addition, immunohistochemical analysis showed that OVA-induced a high level of SYVN1 in the lungs, especially in bronchi (Fig. 1H). Thus, these results suggest that SYVN1 is associated with airway remodeling in OVA-induced asthma.

### SYVN1 overexpression attenuates OVA-induced lung dysfunction

**Fig. 2:**
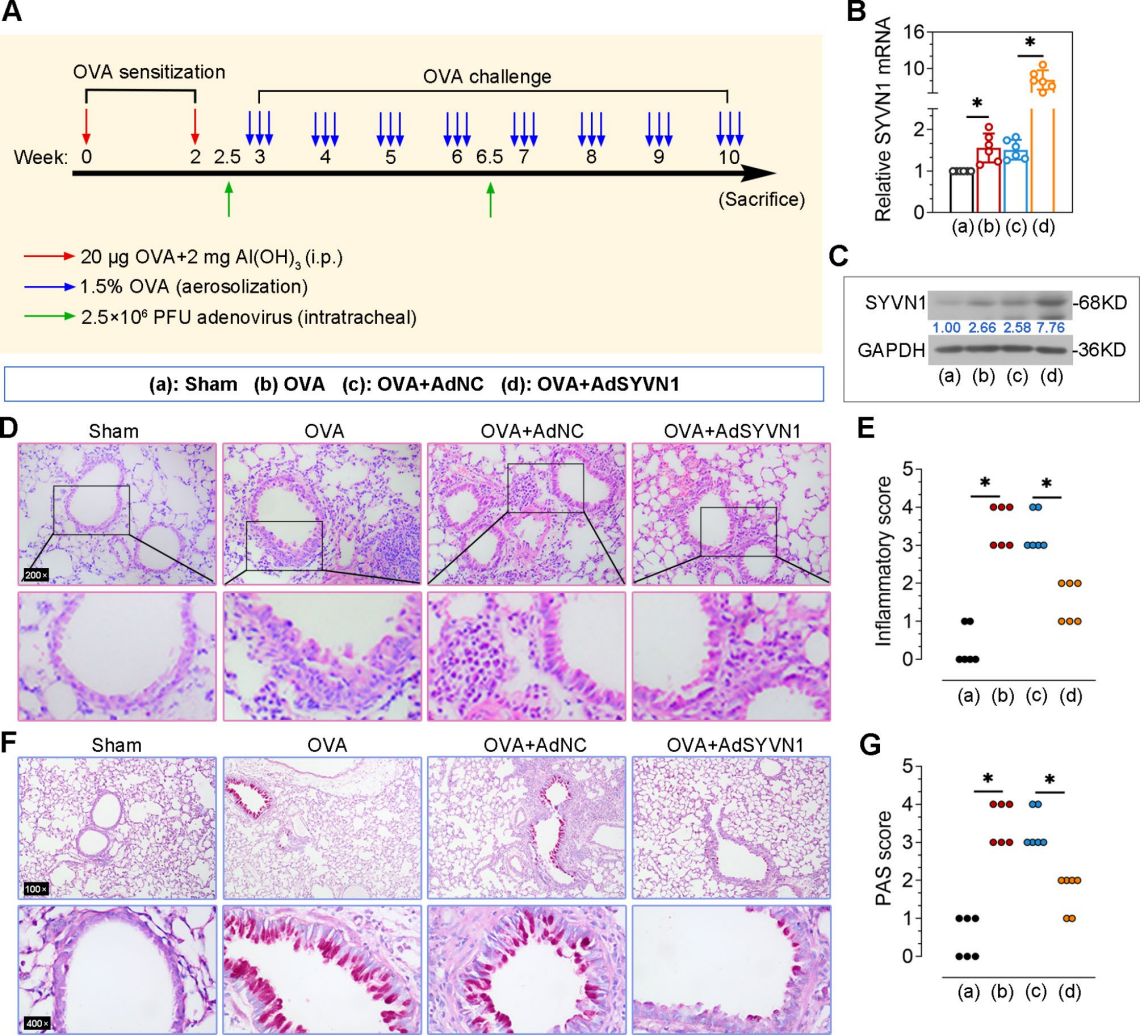
**SYVN1 overexpression attenuates OVA-induced lung dysfunction**. (**A**), Schematic protocol of the OVA-induced asthmatic mice. (**B-C**), The mRNA and protein levels of SYVN1 in the lungs were examined by real-time PCR and western blot. (**D-E**), Representative images of the lung sections stained with H&E. The inflammatory infiltration was scored. (**F-G**), Representative images of the lung sections stained with PAS. PAS scores were quantified to evaluate the goblet cell hyperplasia. *, *P* < 0.05

Adenoviral vectors overexpressing SYVN1 were constructed and infected into mice to study its role in asthma. We found that both the mRNA and protein levels of SYVN1 in the lungs were increased by adenoviral administration (Fig. 2B-C). In addition, results in Fig. 2D-G showed that OVA-induced infiltration of peribronchial inflammatory cells and mucus secretion were inhibited by SYVN1 overexpression. These results demonstrate that SYVN1 has a protective effect on airway inflammation and goblet cell hyperplasia in OVA-induced chronic asthma.

### SYVN1 overexpression prevents OVA-induced collagen deposition and EMT

**Fig. 3:**
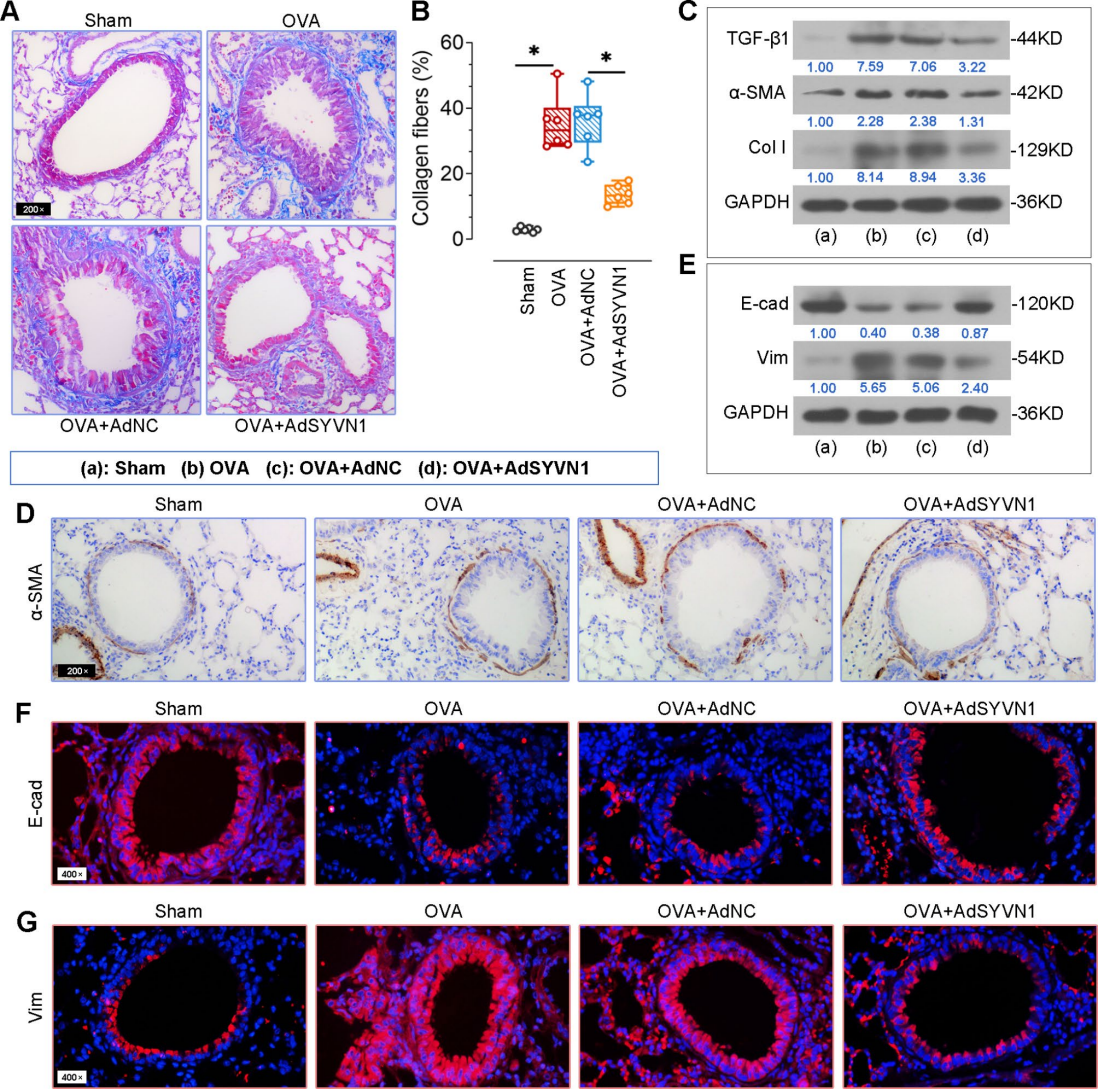
**SYVN1 overexpression prevents OVA-induced collagen deposition and EMT**. (**A**-**B**), Histological analysis of the lung sections stained with Masson. The percentage of collagen fibers were quantified. (**C**), Western blot analysis of TGF-β1, α-SMA, and Collagen I protein expression in the lungs. (**D**), Immunohistochemical examination of α-SMA expression in the lung sections. (**E**), Western blot analysis of E-cadherin and Vimentin protein expression in the lung tissues. (**F-G**), Immunofluorescence analysis of E-cadherin or Vimentin expression in the lung tissues. Epithelial cell marker E-cadherin (red), mesenchymal cell marker Vimentin (red), cell nucleus (DAPI, blue). *, *P* < 0.05

To determine the effect of SYVN1 on collagen deposition in OVA-challenged mice, Masson staining and quantification analysis were performed. As shown in Fig. 3A-B, the increased collagen fibers in the lung tissues were reduced by SYVN1 overexpression. Western blot results also demonstrated that SYVN1 inhibited OVA-induced high expression of fibrotic proteins, including TGF-β1, α-SMA and Collagen I (Fig. 3C). Similar trends of α-SMA expression in lung airways were observed by immunohistochemistry (Fig. 3D). As evidenced by the results of Western blot and immunofluorescence, the decrease in E-cadherin (an epithelial marker) and the increase in Vimentin (a mesenchymal marker) in the lungs of OVA-challenged mice were prevented by SYVN1 overexpression (Fig. 3E-G). Collectively, these results indicate that SYVN1 controls the EMT process in asthma.

### SYVN1 overexpression inhibits OVA-induced ER stress

**Fig. 4:**
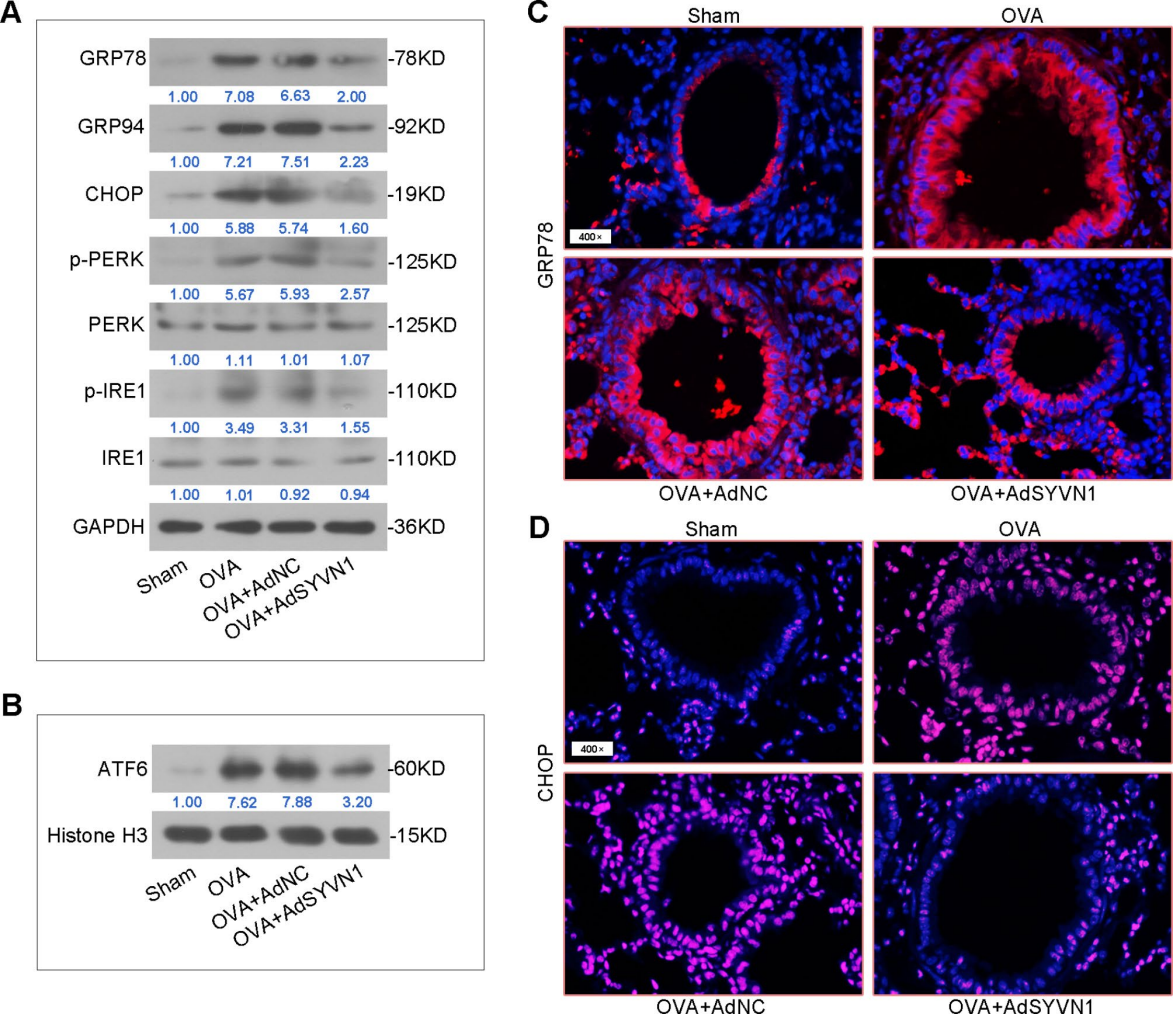
**SYVN1 overexpression inhibits OVA-induced ER stress**. (**A**), Western blot analysis of GRP78, GRP94, CHOP, phosphorylated (p-) PERK, total PERK, p-IRE1 and total IRE1 protein expression in whole cell lysates of the lungs. (**B**), Western blot analysis of ATF6 protein expression in cell nucleus of the lungs. (**C-D**), Immunofluorescence analysis of GRP78 or CHOP expression in the lung sections. GRP78 (red), CHOP (red), cell nucleus (DAPI, blue)

Then, we explored whether SYVN1 is associated with ER stress in OVA-induced asthmatic mice. The unfolded protein responses (UPRs) and activation of ER stress sensing proteins (protein kinase RNA-like endoplasmic reticulum kinase [PERK], inositol-requiring 1 [IRE1] and activating transcription factor 6 [ATF6]) are essential during ER stress. Using Western blot analysis, we found significant increases in GRP78, GRP94, as well as the phosphorylation of PERK (p-PERK) and IRE1 (p-IRE1) in the lungs of OVA-challenged mice (Fig. 4A). OVA administration also increased the protein levels of CHOP (C/EBP homologous protein C/EBP homologous protein) and nuclear ATF6 in the lungs (Fig. 4A-B). Immunofluorescence analysis showed that ER chaperone GRP78 and downstream effector CHOP expression were upregulated in perbronchial cells by OVA (Fig. 4C-D). However, the alterations of ER stress related markers were prevented by SYVN1 overexpression in asthmatic mice. Taken together, these results show an important effect of SYVN1 on ER stress in asthma.

### SYVN1 overexpression suppresses TGF-β1-induced EMT in BEAS-2B cells

**Fig. 5:**
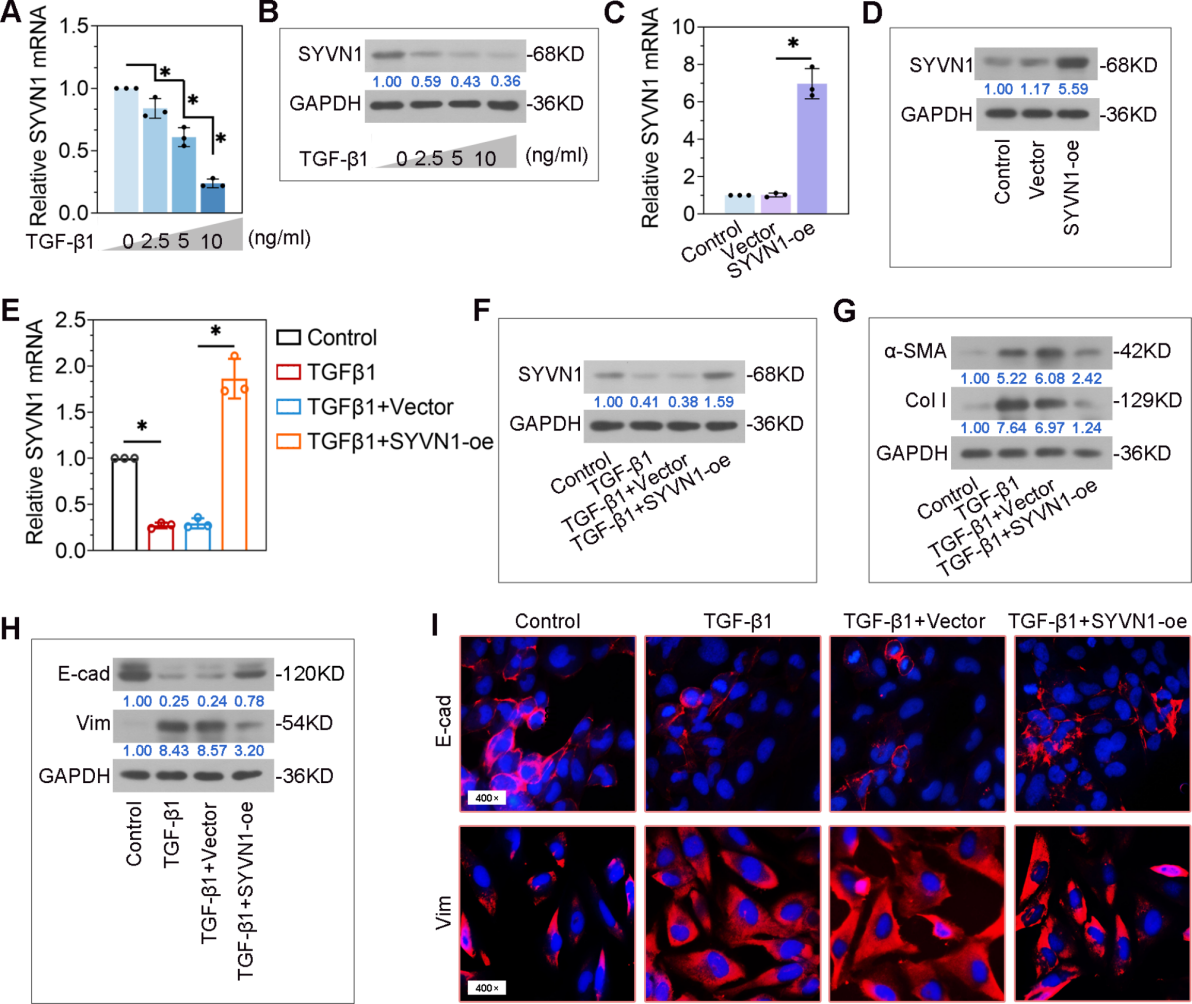
**SYVN1 overexpression suppresses EMT in TGF-β1-induced BEAS-2B cells**. (**A**-**B**), The protein and mRNA expression of SYVN1 were measured using real-time PCR and Western blot in cells stimulated with TGF-β1 at different doses. (**C-D**), The protein and mRNA expression of SYVN1 were determined by real-time PCR and Western blot in cells transfected with empty vector or SYVN1-oe. Cells were transfected with empty vector or SYVN1-oe, followed by TGF-β1 (10 ng/ml) treatment. (**E-F**), The mRNA and protein expression of SYVN1 in cells using real-time PCR and Western blot. (**G**), Western blot analysis of α-SMA and Collagen I protein levels in cells. (**H**), Western blot analysis of E-cadherin and Vimentin protein levels in cells. **I**, Representative immunofluorescence staining of E-cadherin or Vimentin in cells. E-cadherin (red), Vimentin (red), cell nucleus (DAPI, blue). *, *P* < 0.05

TGF-β1 is a critical driver to promote EMT. Thus, TGF-β1-induced BEAS-2B cells were used to examine the role of SYVN1 in EMT process. As shown in Fig. 5A-B, SYVN1 mRNA and protein levels were gradually decreased by TGF-β1 in a dose-dependent manner. Then, the plasmids for SYVN1 overexpression were transfected into BEAS-2B cells (Fig. 5C-D). Real-time PCR and Western blot analysis showed that the decreased expression of SYVN1 in TGF-β1-treated cells were upregulated by its overexpressing vectors (Fig. 5E-F). We found that SYVN1 overexpression caused reductions in α-SMA and Collagen I protein expression in TGF-β1-treated cells (Fig. 5G). Furthermore, TGF-β1-induced decrease in E-cadherin and increase in Vimentin were reversed by SYVN1 (Fig. 5H-I). These in vitro results demonstrate that SYVN1 regulates the EMT process in TGF-β1-treated bronchial epithelial cells.

### ER stress is involved in the effect of SYVN1 on TGF-β1-induced EMT in BEAS-2B cells

**Fig. 6:**
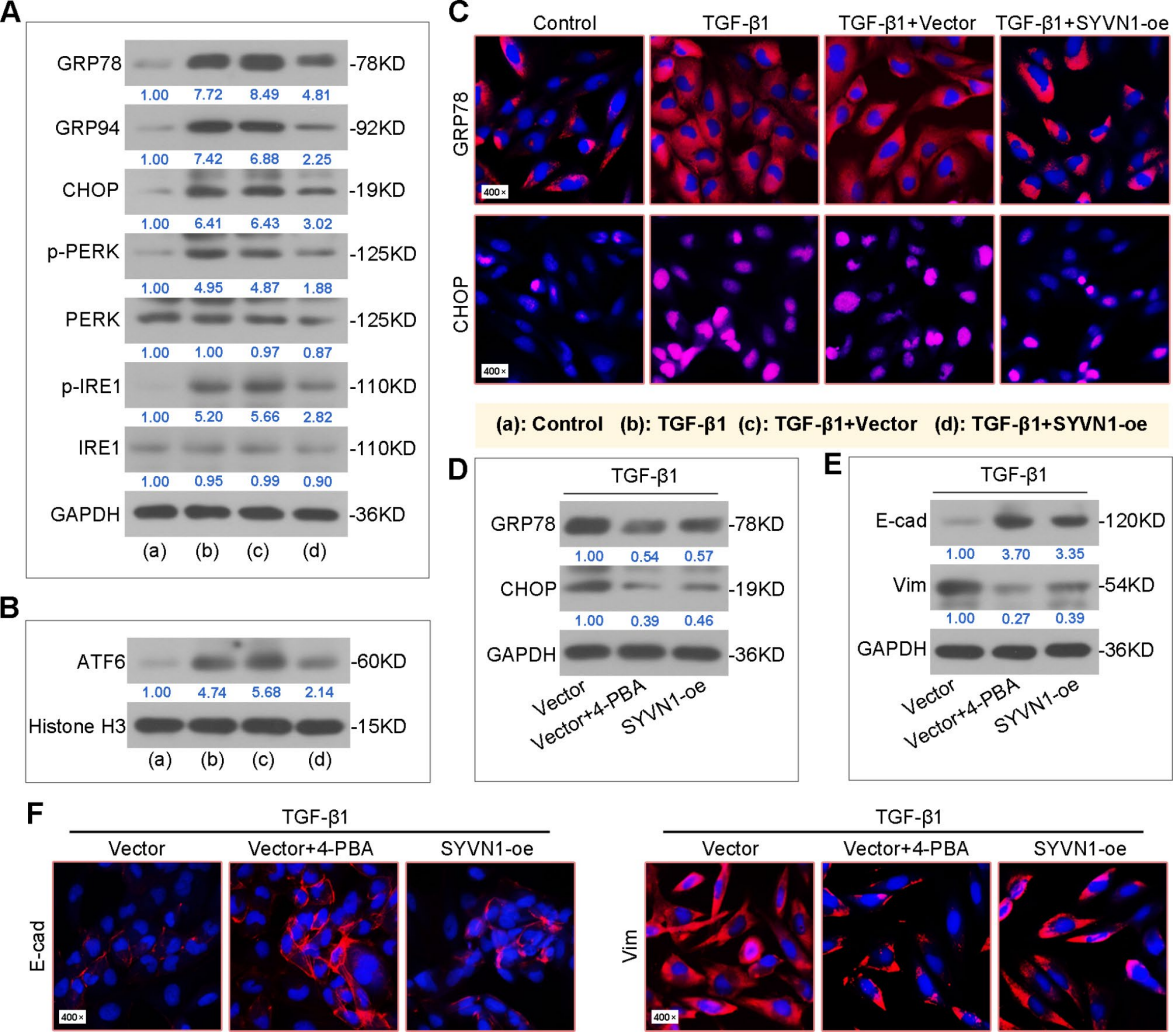
**ER stress is involved in the effect of SYVN1 on EMT in TGF-β1-induced BEAS-2B cells**. (**A**), Western blot analysis of GRP78, GRP94, CHOP, p-PERK, total PERK, p-IRE1 and total IRE1 protein expression in whole cell lysates. (**B**), Western blot analysis of ATF6 protein expression in cell nucleus. (**C**), Representative immunofluorescence images of cells stained with GRP78 or CHOP. GRP78 (red), CHOP (red), cell nucleus (DAPI, blue). (**D**), Western blot analysis of GRP78 and CHOP protein expression in cells. (**E**), Western blot analysis of E-cadherin and Vimentin protein expression in cells. (**F**), Representative immunofluorescence staining of E-cadherin or Vimentin. E-cadherin (red), Vimentin (red), cell nucleus (DAPI, blue)

For the examination of ER stress markers in vitro, we found that SYVN1 overexpression reduced TGF-β1-induced increases in GRP78, GRP94, CHOP, p-PERK and p-IRE1 protein expressions in whole cell lysates as well as ATF6 protein expression in nucleus by western blot (Fig. 6A-B). Immunofluorescence staining also demonstrated similar trends of GRP78 and CHOP expression (Fig. 6C). Thus, the ER stress inhibitor 4-phenylbutyrate (4-PBA) was used to evaluate the implication of ER stress in TGF-β1-induced EMT. As shown in Fig. 6D, the protein levels of GRP78 and CHOP in TGF-β1-treated cells were suppressed by 4-PBA treatment or SYVN1 overexpression. In addition, we observed that, similarly to the effect of 4-PBA, SYVN1 overexpression also increased E-cadherin and decreased Vimentin levels by western blot and immunofluorescence staining (Fig. 6E-F). These results imply an involvement of ER stress in SYVN1’s effect on TGF-β1-induced EMT.

### SYVN1 interacts with SIRT2 to promote its ubiquitylation and degradation

**Fig. 7:**
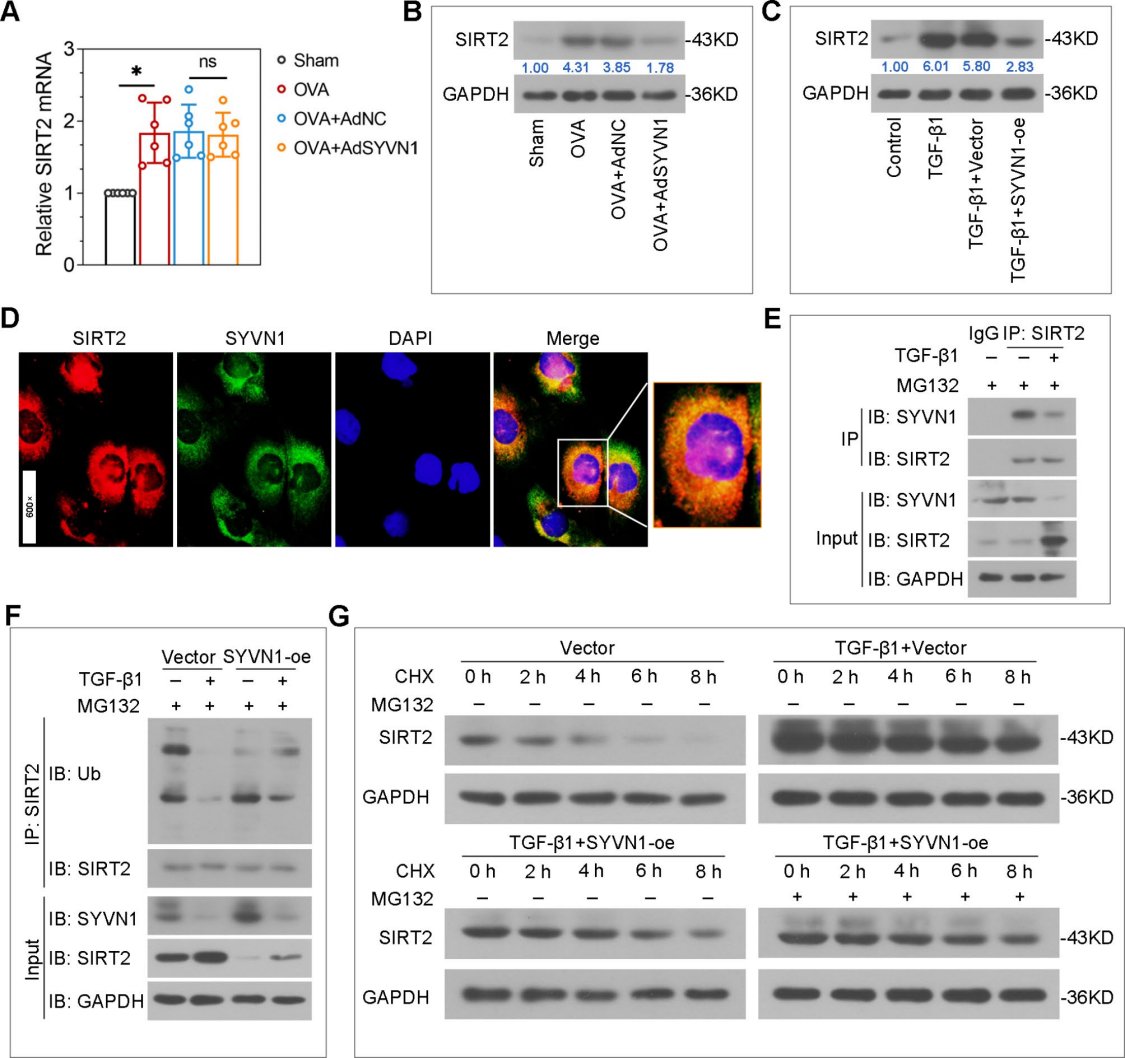
**SYVN1 interacts with SIRT2 to promote its ubiquitylation and degradation**. (**A**-**B**), The mRNA and protein expression of SIRT2 in the lung tissues were measured by real-time PCR and Western blot. (**C**), Western blot analysis of SIRT2 protein expression in BEAS-2B cells. (**D**), The cellular colocalization of SIRT2 and SYVN1 in BEAS-2B cells were examined by immunofluorescence staining. SIRT2 (red), SYVN1 (green), cell nucleus (DAPI, blue), merge (SIRT2 + SYVN1 + DAPI). (**E**), Immunoprecipitation and Western blot analysis of BEAS-2B cells, followed by IP with anti-SIRT2. (**F**), Immunoprecipitation and Western blot analysis of SIRT2 ubiquitylation in BEAS-2B cells. (**G**), Western blot analysis of SIRT2 protein expression in BEAS-2B cells treated with cycloheximide (CHX; 50 µg/ml) or MG132 (10 µM) at various time points. *, *P* < 0.05; ns, no significance

SIRT2 is shown to be a contributor of airway inflammation in allergic asthma. Results in Fig. 7A-B suggested that SYVN1 overexpression significantly inhibited SIRT2 protein level in the lungs of OVA-induced mice, rather than the mRNA level. In vitro, TGF-β1-induced upregulation of SIRT2 protein were also reduced by SYVN1 (Fig. 7C). To determine the regulatory mechanism of SYVN1 on SIRT2, we first demonstrated that both endogenous SYVN1 and SIRT2 were colocalized in BEAS-2B cells using immunofluorescence staining (Fig. 7D). Co-IP results showed that SYVN1 specifically interacted with SIRT2 in BEAS-2B cells, and the interaction between SYVN1 and SIRT2 was reduced by TGF-β1 treatment (Fig. 7E). Next, we investigated whether SYVN1 regulates the ubiquitination and degradation of SIRT2 via ubiquitination-proteasome system. Our results indicated that overexpression of SYVN1 significantly increased SIRT2 ubiquitination, and stimulation of TGF-β1 prevented SIRT2 ubiquitination (Fig. 7F). In addition, the extended half-life of SIRT2 in TGF-β1-induced cells were suppressed by SYVN1 overexpression, while SIRT2 protein expression was restored in the presence of MG132 (a proteasome inhibitor) (Fig. 7G), suggesting that SYVN1 regulated SIRT2 protein stability through proteasome pathway. Taken together, these results demonstrate that SYVN1 mediates the ubiquitination and degradation of SIRT2.

### SYVN1 affects ER stress and EMT by the regulation of SIRT2 in TGF-β1-induced BEAS-2B cells

**Fig. 8:**
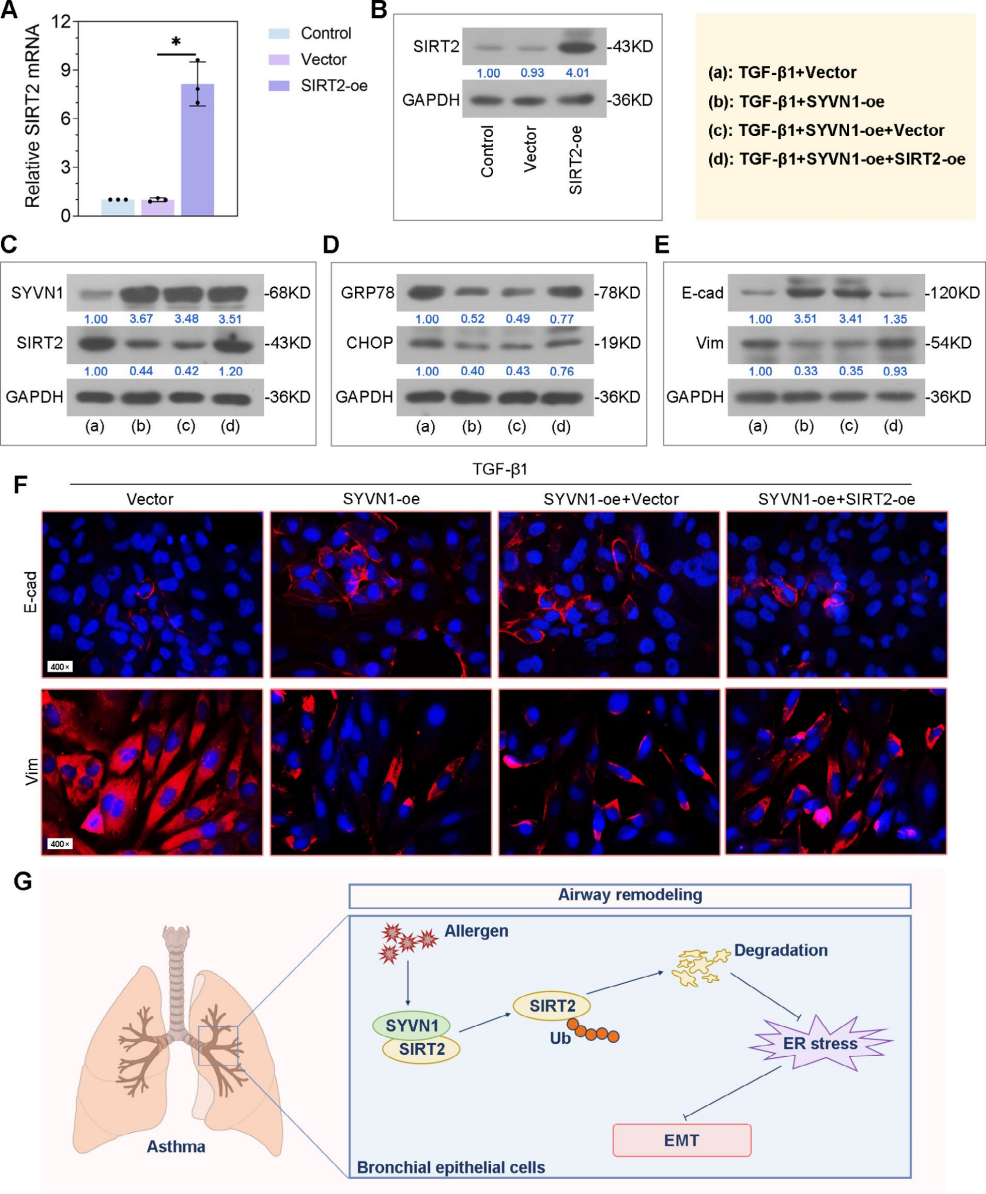
**SYVN1 affects ER stress and EMT by the regulation of SIRT2 in TGF-β1-induced BEAS-2B cells**. (**A**-**B**), The mRNA and protein expression of SIRT2 in cells transfected with empty vector or SIRT2-oe. Cells were cotransfected with Vector/SYVN1-oe together with Vector/SIRT2-oe, and then treated with TGF-β1. (**C**), Western blot analysis of SYVN1 and SIRT2 protein expression in cells. (**D**), Western blot analysis of GRP78 and CHOP protein expression in cells. (**E**), Western blot analysis of E-cadherin and Vimentin protein expression in cells. (**F**), Representative immunofluorescence images of cells stained with E-cadherin and Vimentin. E-cadherin (red), Vimentin (red), cell nucleus (DAPI, blue). (**G**), Schematic diagram for the role of SYVN1 in airway remodeling in asthma. *, *P* < 0.05

Then, we evaluated whether SIRT2 mediates the regulation of SYVN1 on ER stress and EMT induced by TGF-β1. SIRT2 overexpression plasmids were constructed and transfected into BEAS-2B cells treated with TGF-β1 (Fig. 8A-B). Overexpression of SIRT2 caused an increase in SIRT2 protein expression in TGF-β1-treated cells together with SYVN1-oe transfection, rather than SYVN1 protein expression (Fig. 8C). In addition, the inhibition of GRP78 and CHOP by SYVN1 overexpression was restored by SIRT2 in cells treated with TGF-β1 (Fig. 8D). Using western blot and immunofluorescence staining, we found that SIRT2 overexpression reversed the suppression of SYVN1 on EMT process in TGF-β1-induced cells (Fig. 8E-F). These results indicated that SIRT2 mediates the inhibitory impact of SYVN1 on ER stress and EMT in TGF-β1-induced bronchial epithelial cells.

## Discussion

Airway remodeling is a critical structural change with complex pathogenesis in asthma. In the present study, we demonstrated the high expression of SYVN1 in OVA-induced asthmatic mice. Interestingly, SYVN1 was decreased in bronchial epithelial cells with TGF-β1 stimulation. Overexpression of SYVN1 mitigated airway inflammation, collogen deposition, EMT and ER stress by OVA challenge. Mechanistic studies showed that SYVN1 promoted SIRT2 ubiquitination and degradation to suppress ER stress, leading to the prevention of EMT in bronchial epithelial cells.

Several pathogens, cigarette smoke and allergens (such as OVA and house dust mite) are the major components to elicit airway inflammation and remodeling [14]. Animals sensitized and challenged with OVA are well-established to develop pathophysiological symptoms similar to human asthma, including airway inflammation, goblet cell hyperplasia, smooth muscle cell hypertrophy and subepithelial fibrosis [15]. In the present study, inflammatory cell infiltration, mucus production and collagen deposition by OVA induction were abrogated by SYVN1 overexpression. Although a 2.73-fold increase in SYVN1 expression was observed in OVA-induced mice, a more significant upregulation of SYVN1 (7.76 folds) were found by its overexpressing adenovirus. The functional experiments demonstrated that SYVN1 might mediate a protective mechanism on airway remodeling in chronic asthma. We thought one reason is that the underlying mechanisms of asthma is clearly very complex. The body produces a number of protective factors to fight the pathological changes in asthma. Several protective factors are highly expressed in the asthmatic model, whereas they are still hard to display a protective effect due to the limited repair ability of body.

ER stress acts as a central regulator in maintaining cellular function, calcium homeostasis and cell signaling, and it is defined as the accumulation of unfold/misfold proteins in ER [16]. Prolonged UPRs are sensed by the three transmembrane proteins of PERK, IRE1 and ATF6, subsequently induced an upregulation of CHOP [17]. Several allergens have been reported to trigger ER stress and disturb ER homeostasis [18, 19]. The presence of ER stress has been demonstrated by changes in GRP78 and CHOP expression in the lungs of patients with asthma [20–22]. ER stress development and unfolded protein response (UPR) activation in airway epithelial cells or inflammatory cells contribute to the induction and maintenance of asthma, such as airway inflammation and fibrosis [19, 21]. Studies in animals suggest that blocking ER stress (such as 4-PBA) attenuates airway inflammation and remodeling in asthma [7, 23]. To our knowledge, SYVN1 is an ER-associated degradation-associated E3 ubiquitin ligase that functions by promoting unfold/misfold protein degradation in ER [6]. Xu et al. suggest that SYVN1 is induced by ER stress response, and it in turn degrades the expression of ER stress genes [24], which indicating a feedback loop between ER stress and SYVN expression. Our results showed that the ER stress in OVA-induced asthma mice was abrogated by SYVN1 overexpression. Thus, we thought that the activation of ER stress in asthma partially caused an increase in SYVN1 expression, and the SYVN1 suppressed ER stress response to protect against airway remodeling.

It is reported that epithelial cells are one of the major cell types in asthma, and these cells transformed into mesenchymal phenotypes have critical impacts on airway remodeling [25]. The EMT process can induce organ fibrosis, tissue repair and cancer metastasis [26, 27]. Suppression of EMT is demonstrated to alleviate airway hyperresponsiveness and fibrosis [28]. TGF-β1 is a well-known cytokine that contributes to EMT and collagen deposition [29]. In addition, studies have revealed that SYVN1 expression is downregulated by TGF-β1 stimulation, and knockdown of SYVN1 promotes EMT progress in tubular epithelial cells [30]. Fan et al. suggest that SYVN1 functions as a negative regulator of the EMT process in breast cancer [31]. Consistent with these findings, our results showed that SYVN1 abrogated EMT and collagen deposition in OVA-induced asthma mice and TGF-β1-induced bronchial epithelial cells. However, the underlying mechanism of SYVN1 on EMT in asthma is unclear.

Emerging evidence has reported that ER stress response in injured lungs causes epithelial cell dysfunction, which promotes fibrotic remodeling [32]. Hoffman et al. show that inhibiting ER stress protects against airway inflammation and fibrosis in chronic asthma [9]. In this study, we found that both SYVN1 overexpression and chemical inhibition of ER stress prevented EMT process and reduced collagen deposition in vitro. In addition, the ubiquitination-proteasome system participates in various biological processes through degrading target proteins [33, 34]. SYVN1 is an E3 ubiquitination ligase that mainly located in the ER, and it is shown to decrease SIRT2 expression in HEK293T cells by promoting its ubiquitination and degradation [13]. In our study, the direct interaction between SYVN1 and SIRT2 in bronchial epithelial cells was also confirmed through the ubiquitination and degradation, and TGF-β1 treatment reduced this interaction. Accumulating studies indicate that SIRT2 can activate ER stress response, and it mediates the exacerbation of allergic asthma [11, 35]. Here, we found that TGF-β1 significantly upregulated SIRT2 expression in BEAS-2B cells, and SYVN1 overexpression induced a decrease in SIRT2. Overexpression of SIRT2 reversed the effect of SYVN1 on ER stress, collagen deposition and EMT in vitro.

## Conclusions

Collectively, our results suggest that SYVN1 promotes SIRT2 ubiquitination and induces inactivation of ER stress, which blocks EMT process and attenuates airway remodeling in asthma.

## Methods

### Animal model of chronic Asthma

All animal experiments were performed according to the Guide for the Care and Use of Laboratory Animals, and approved by the ethical committee of Shengjing Hospital of China Medical University. Female C57BL/6 mice at the age of 6 weeks were purchased from Changsheng (China). Chronic asthmatic model was induced as the previous described [36]. Briefly, the mice were sensitized with 20 µg ovalbumin (OVA; Aladdin, China) and 2 mg Al(OH)_3_ by two intraperitoneal injections on days 0 and 14. From days 21, mice were challenged with 1.5% aerosolized OVA for 45 min. The OVA aerosol challenge was conducted 3 times/week (every other day, a total of 8 weeks). Twenty-four hours following the last challenge, mice were sacrificed. The controls (Sham) were sensitized and challenged with saline. Adenovirus expressing SYVN1 (AdSYVN1) or negative control (AdNC) (2.5 × 10^6^ PFU) was prepared to intratracheal injection for 3 days before the first aerosol. Four weeks later, the adenovirus delivery was repeated. The schematic timeline for animal protocols is shown in Fig. 2A.

### Construction of recombinant adenovirus

To construct the recombinant adenovirus expressing SYVN1, the SYVN1 gene sequences were synthesized by General Bio (China) and inserted into the NotI and HindIII sites of pShuttle-CMV vectors (Fenghui, China). The plasmids were transfected into HEK-293 A cells (iCell, China) using Lipofectamine 3000 (Invitrogen, USA) to produce the recombinant adenovirus.

### Lung histopathology

Upon sacrifice, the lung tissues were fixed in 4% paraformaldehyde and embedded in paraffin. Then, the paraffin-embedded lung Sect. (5-µm thick) were subjected to Hematoxylin and eosin (H&E), periodic acid-Schiff (PAS) and Masson staining as standard protocols to determine inflammation, mucus metaplasia and collagen deposition. The morphological photographs were observed under the BX53 microscope (Olympus, Japan) equipped with a DP73 Olympus camera (Olympus, Japan) via 10×, 20×, or 40× objective lenses. Images were obtained using CellSens software (Olympus, Japan) and analyzed using Image-Pro Plus 6.0 software (Media Cybernetics, USA).

The inflammatory score was assessed using the method reported by Cho et al. [37]. In brief, inflammation was scored as following: score 0 (no inflammatory cells were detectable), score 1 (occasional observation of inflammatory cells), score 2 (bronchi or vessels were surrounded by 1–3 layers of inflammatory cells), score 3 (bronchi or vessels were surrounded by 4–5 layers of inflammatory cells), and score 4 (bronchi or vessels were surrounded by more than 5 layers of inflammatory cells).

The goblet cell hyperplasia was scored according to the previous study based on the ratio of goblet cells in the epithelium [38]: score 0 (no goblet cells), score 1 (< 25% goblet cells), score 2 (25–50% goblet cells), score 3 (51–75% goblet cells), and score 4 (> 75% goblet cells).

To assess collagen deposition, the staining area (blue) and total area were quantified and the percentage of collagen fibers were calculated as the collagen area (blue)/total area as previously reported [39].

### Immunohistochemical staining

For immunohistochemical staining, the paraffin-embedded sections of lung tissues were incubated with the α-smooth muscle actin (α-SMA) antibody (Affinity, China) or SYVN1 antibody (Proteintech, China) overnight at 4 °C. Then, the HRP-conjugated goat anti-rabbit antibody (ThermoFisher, USA) was used to incubate for 60 min at 37 °C. The results were visualized by the BX53 microscope (Olympus, Japan) equipped with a DP73 Olympus camera (Olympus, Japan) via 20× objective lenses and obtained using CellSens software (Olympus, Japan).

### Cell culture and treatment

BEAS-2B cells (iCell, China) were cultured in Dulbecco’s Modified Eagle’s Medium (DMEM) containing 10% fetal bovine serum (FBS) in an incubator with 5% CO_2_ condition at 37 °C. To induce epithelial-mesenchymal transition, the cells were treated with TGF-β1 (SinoBiological, China) at the dose of 2.5, 5 or 10 ng/ml for 24 h.

To overexpress SYVN1 or SIRT2 expression, the plasmids for targeting SYVN1 (SYVN-oe), SIRT2 (SIRT2-oe) or negative control (Vector) were transfected into BEAS-2B cells for 48 h using Lipofectamine 3000 (Invitrogen, USA), according to the manufacturer’s protocol. In addition, the ER stress inhibitor 4-PBA (5 mM; MACKLIN, China) was administrated to the cells for 2 h prior to TGF-β1 treatment.

### Co-immunoprecipitation (Co-IP)

Co-IP assay was carried out using a Pierce Co-IP kit (ThermoFisher, USA) according to the manufacture’s protocol. Cells were pretreated with 10 µM MG132 (Aladdin, China) for 6 h, and then were collected and lysed with an immunoprecipitation buffer (Beyotime, China) to extract proteins. The extracts were incubated with the AminoLink Plus Coupling Resin along with the immobilized SIRT2 antibody (Santa Cruz, USA). After incubation, the immune complex was washed and determined by Western blot.

### Immunofluorescence staining

The paraffin-embedded lung sections and fixed cells were conducted to immunofluorescence staining. In brief, the tissue slides or cells were incubated with primary antibodies against E-cadherin (Affinity, China), Vimentin (Affinity, China), GRP78 (Proteintech, China), CHOP (Affinity, China) overnight at 4 °C, followed by the incubation of Cy3-conjugated anti-rabbit (Invitrogen, USA) or Cy3-conjugated anti-mouse (Invitrogen, USA) for 60 min at room temperature. Photographs were captured under the BX53 microscope (Olympus, Japan) equipped with a DP73 Olympus camera (Olympus, Japan) via 40× objective lenses (blue filter for DAPI, red filter for GRP78, CHOP, E-cadherin and Vimentin) using CellSens software (Olympus, Japan). In addition, primary antibodies against SYVN1 (Proteintech, China) and SIRT2 (Santa Cruz, USA) were used to incubate with cells overnight at 4 °C to identify the colocalization of SYVN1 and SIRT2 in cells. Then, the FITC-conjugated anti-rabbit (Abcam, UK) and Cy3-conjugated anti-mouse (Invitrogen, USA) secondary antibodies were added and incubated for 60 min at room temperature. Images were taken with a laser scanning confocal microscope (C2, Nikon, Japan) via 60× objective lenses (blue filter for DAPI, red filter for SIRT2, green filter for SYVN1).

### Real-time PCR

Total RNA was extracted using the TRIpure reagent (Bioteke, China) and was reverse transcribed to cDNA using a BeyoRT II M-MLV Reverse Transcriptase (Beyotime, China). Real-time PCR was carried out using the specific primers (Table  1) and SYBR Green probes (Solarbio, China) with the Exicycler^96^ Real-time PCR system (Bioneer, Korea). The GAPDH expression was used as an internal control. The 2^−ΔΔCt^ method was used to obtain mRNA levels.

### Western blot

The lung tissues and cells were lysed with a RIPA buffer (Solarbio, China) supplemented a protease inhibitor. After quantification, protein extracts were subjected to SDS-PAGE and transferred to PVDF membranes. The membranes were blocked with 5% non-fat milk for 1 h and incubated with primary antibodies overnight at 4 °C, including SYVN1 antibody (Proteintech, China), SIRT2 antibody (Affinity, China), α-SMA antibody (Affinity, China), TGF-β antibody (Affinity, China), collagen I antibody ((Affinity, China), E-cadherin antibody (Affinity, China), Vimentin antibody (Affinity, China), GRP94 antibody (Affinity, China), GRP78 antibody (Proteintech, China), CHOP antibody (Affinity, China), p-PERK antibody (Affinity, China), PERK (Affinity, China), p-IRE antibody (Affinity, China), IRE antibody (Affinity, China), ATF6 antibody (Proteintech, China), Ubiquitin (linkage specific K48) antibody (Abcam, UK), Histone antibody (Proteintech, China) and GAPDH antibody (Proteintech, China). Subsequently, the HRP-conjugated anti-rabbit (Solarbio, China) and HRP-conjugated anti-mouse (Solarbio, China) secondary antibodies were used for incubation for 1 h at 37 °C. The protein bands were visualized using ECL chemiluminescence solution (Solarbio, China). The gray values were analyzed using Gel-Pro-Analyzer software (Media Cybernetics, USA). The target bands were standardized to the internal control and then normalized against the mean of protein level in the control group.

### Statistical analysis

Data were shown as mean ± SD, and data difference analysis was performed using GraphPad Prism 8.0. Comparisons between two groups were analyzed by t test. One-way ANOVA following Bonferroni’s test was conducted for multiple comparisons. The statistical significance was identified when *p* < 0.05.

## Data Availability

The data supporting this study’s findings are available from the corresponding author upon reasonable request.

## List of Abbreviations

α-SMA: α-smooth muscle actin
Ad: Adenovirus
ATF6: Activating transcription factor 6
CHOP: C/EBP homologous protein C/EBP homologous protein
DMEM: Dulbecco’s Modified Eagle’s Medium
EMT: Epithelial-mesenchymal transition
ER: Endoplasmic reticulum
H&E: Hematoxylin and eosin
IRE1: Inositol-requiring 1
NC: Negative control
OVA: Ovalbumin
PAS: Periodic acid-Schiff
PERK: Protein kinase RNA-like endoplasmic reticulum kinase
SIRT2: Sirtuin 2
SYVN1: Synoviolin 1
